# Nasal mucus glutathione transferase activity and impact on olfactory perception and neonatal behavior

**DOI:** 10.1038/s41598-019-39495-6

**Published:** 2019-02-28

**Authors:** Aline Robert-Hazotte, Philippe Faure, Fabrice Neiers, Catherine Potin, Yves Artur, Gérard Coureaud, Jean-Marie Heydel

**Affiliations:** 1 Centre des Sciences du Goût et de l′Alimentation, UMR 6265 CNRS/1324 INRA/Université de Bourgogne Franche-Comté, 9 boulevard Jeanne d’Arc, F-21000 Dijon, France; 2 Centre de Recherche en Neurosciences de Lyon, INSERM U1028/CNRS UMR 5292/Université Lyon 1, CH Le Vinatier-Bâtiment 462 Neurocampus, 95 boulevard Pinel, F-69675 Bron, France

**Keywords:** Transferases, Neurophysiology

## Abstract

In olfaction, to preserve the sensitivity of the response, the bioavailability of odor molecules is under the control of odorant-metabolizing enzymes (OMEs) expressed in the olfactory neuroepithelium. Although this enzymatic regulation has been shown to be involved in olfactory receptor activation and perceptual responses, it remains widely underestimated in vertebrates. In particular, the possible activity of OMEs in the nasal mucus, i.e. the aqueous layer that lined the nasal epithelium and forms the interface for airborne odorants to reach the olfactory sensory neurons, is poorly known. Here, we used the well-described model of the mammary pheromone (MP) and behavioral response in rabbit neonates to challenge the function of nasal mucus metabolism in an unprecedented way. First, we showed, in the olfactory epithelium, a rapid glutathione transferase activity toward the MP by *ex vivo* real-time mass spectrometry (PTR-MS) which supported an activity in the closest vicinity of both the odorants and olfactory receptors. Indeed and second, both the presence and activity of glutathione transferases were evidenced in the nasal mucus of neonates using proteomic and HPLC analysis respectively. Finally, we strikingly demonstrated that the deregulation of the MP metabolism by *in vivo* mucus washing modulates the newborn rabbit behavioral responsiveness to the MP. This is a step forward in the demonstration of the critical function of OMEs especially in the mucus, which is at the nasal front line of interaction with odorants and potentially subjected to physiopathological changes.

## Introduction

Olfaction strongly contributes to the adaptation and welfare of animals - including humans - all over their development. Indeed, it directly influences behavioral actions that allow responding to vital needs, e.g., social interaction, partners’ choice, mother-young relationships, food searching, predator avoidance. However, certain biochemical mechanisms that support the efficient processing of odor molecules have been only recently highlighted and remain to be fully elucidated due to their suspected involvement in odor perception and resulting behaviors. For instance, the function of odorant-metabolizing enzymes (OMEs) in the processing of odorants at the periphery of the olfactory system known a growing interest in vertebrates because these enzymes, highly expressed in the olfactory tissues, turn out to play a significant role in olfaction. As members of the xenobiotic-metabolizing enzymes family, OMEs have been initially involved in the clearance and deactivation of the odorants from the perireceptor environment, which are necessary to maintain the sensitivity of odorant detection by the olfactory receptors^1^. This is achieved through two enzymatic steps involving different classes of OMEs. Phase I enzymes like cytochromes P450 (CYP) or carboxylesterases catalyze the biotransformation of apolar odorants resulting in phase I metabolites carrying functional groups (OH, NH_2_, COOH). Phase II enzymes, like UDP-glucuronosyltransferases (UGT), glutathione transferases (GST) or sulfotransferases can take in charge either odorants naturally functionalized or phase I metabolites by catalyzing their conjugation with big polar moiety (glucuronic acid, glutathione, sulfate). The phase II step, leading to hydrophilic forms of the initial odorants molecules, has been logically involved in the termination of the peripheral olfactory signal^2–4^. Conversely, recent studies have suggested that phase I participates to the modulation of the olfactory signal through synthesis^5,6^ of active phase I metabolites^7–9^. The phase III involves transporter proteins for the extracellular excretion of metabolites.

In mammals, the action and biological influence of OMEs have been studied in the rabbit, as a model, since several years. Lactating rabbit females produce in their milk a particular odor molecule, the mammary pheromone (MP; 2-methylbut-2-enal) which elicits very strong and specific orocephalic movements in newborns that help them to locate rapidly the nipples and suck. Since the mother nurses only once and briefly per day, it is critical for the pups to remain sensitive to the MP during the 5 min of maternal presence^10–15^. Interestingly, GST appear to be involved in MP processing in rabbit neonates and behavioral responsiveness to this maternal signal. Indeed, we first demonstrated a high GST (phase II enzymes) activity toward the MP in the olfactory mucosa (OM) of newborn compared to weanling rabbits (at weaning the typical orocephalic movements are no longer displayed to the MP)^16^. This suggested a direct function of GSTs in the active clearance of the MP from the perireceptor environment in this developmental period where a high sensitivity to the MP must be maintained to ensure survival^11,13^.We further developed an *ex vivo* headspace gas-chromatography method to measure MP metabolism in *quasi* physiological exposure conditions, i.e., by suing intact OM fresh explants in contact with MP on gaseous form. The results confirmed the efficient MP metabolizing activity in newborn OM^17^. Since the headspace method allowed measuring the metabolism of odorants in mixtures, we tested if, as observed in drug metabolism, competitive metabolic interaction could arise between two distinct odorants, by modifying their relative perireceptor availability. Clearly, the MP metabolism was significantly modified in presence of another aldehyde (2MP2; 2-methylpent-2-enal) which increased MP concentration in the receptor environment^18^. The final consequence was that the metabolic competition between MP and 2MP2 significantly exhausted the perception of the MP in rabbit pups probably as a result of a larger number of activated receptors. Indeed, newborns responded to the MP in mixture with 2MP2 at a concentration which constitutes a sub-detection threshold when MP is presented alone^18^. For all these reasons, the newborn rabbit and MP currently constitute an unprecedented model in vertebrates to investigate the function of phase II enzymes in olfactory perception, behavior and adaptation.

In most of the studies on OMEs function, the enzymatic activity was measured in OM homogenates or explants which encompass the different epithelial cellular types as well as the olfactory mucus. If the mucus has been classically considered as a nasal aqueous interface dissolving odor molecule *prior* to their detection by the olfactory receptor neurons, recent findings lead to assume that it could also be the place of enzymatic regulation of these molecules with a direct impact on their bioavailability. Accordingly, a study in mice showed that the nasal mucus exhibited phase I carboxylesterases activity toward odorants^8^ but the corresponding enzyme expression was not evidenced. Conversely, the presence of phase II GST enzymes in the human olfactory mucus was suggested by proteomic analysis^19^ but the corresponding activity has never been recorded. The effective presence of OMEs in the mucus, in the immediate vicinity of odorants and olfactory receptors would strongly support their role in the fast treatment of the peripheral signal. In the present study, our goal and challenge were to study the GST velocity and enzymatic activity (to date not clearly evidenced) toward the MP in the OM and nasal mucus of the newborn rabbit. In this purpose, we used here a previously developed and very sensitive mass spectrometry method based on PTR-Tof-MS (PTR-MS) instrument^5^ to monitor, in real-time, the *ex vivo* olfactory metabolism of the MP. The GST activity toward the MP was measured in the nasal mucus using HPLC coupled with charged aerosol detector (HPLC-CAD). Complementarily, the presence and identification of GST in the nasal mucus of neonates was investigated by proteomic analysis. Finally, the newborn rabbits’ sucking-related behavior was tested in order to determine the influence of the nasal mucus, supposed to be containing enzymes, in the perception of the MP.

## Results

### Real-time *ex vivo* olfactory metabolism of MP by the newborn rabbit OM

**Figure 1:**
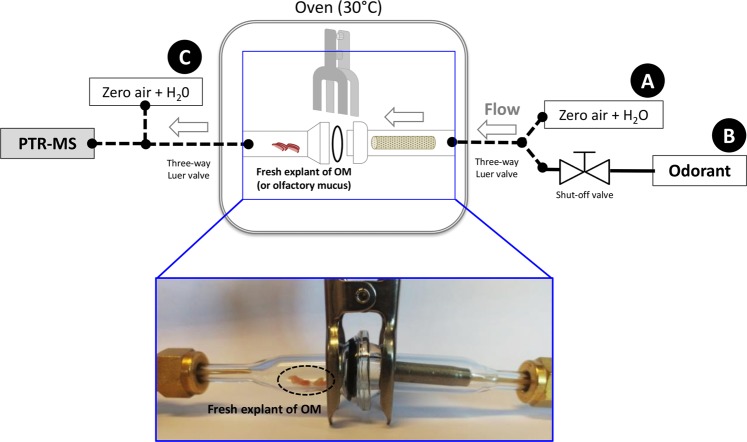
Schematic representation of the system developed to analyse the metabolic capacity of *ex vivo* newborn rabbit OM or nasal mucus by PTR-MS in real-time. A known concentration of gaseous odorants (gas bag B) can be delivered above a fresh explant of OM or nasal mucus placed into a hermetically closed glassware and the flow was monitored by the PTR-MS instrument allowing the real-time analysis of metabolic activities. Shut-off valve and three-way valve luer were implemented to allow delivery of humidified zero air passing through the glassware (gas bag A) or not (gas bag C) to realize analysis and controls in different conditions. The whole system is enclosed in a thermostated oven (30 °C).

**Figure 2:**
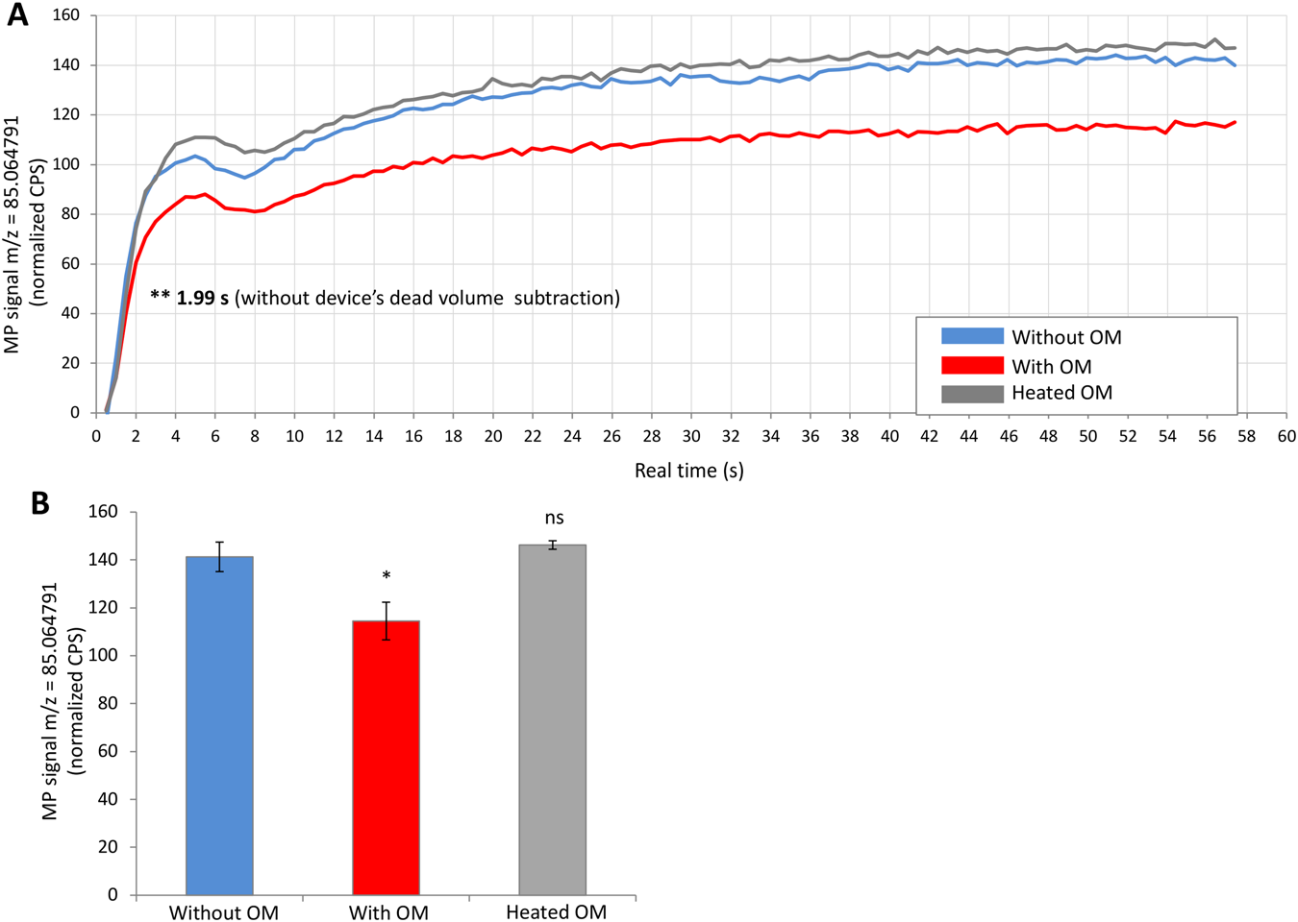
Real-time *ex vivo* newborn rabbit OM metabolism of the MP by PTR-MS analysis with the continuous method. The signal of MP on gaseous form (10^−9^ g/ml in gas bag B) passing through the glassware without OM (in blue), with OM (in red) or in presence of heated OM to denaturate enzymes (in grey) was monitored in real-time by the PTR-MS using the continuous method. (**A**) Kinetics of the *ex vivo* MP metabolism was measured in continuous over 60 s. Data represent the mean ± SEM from 5 independent assays for each condition. The significant differences is noted ^**^p < 0.01 (Student’s t-test) for a comparison with MP signal without OM. (**B**) Data represent the normalized CPS mean ± SEM during the last 20 s of the continuous supply signal of MP measured by the PTR-MS instrument. No significant difference is noted ns and significant difference is noted ^*^p < 0.05 (Student’s t-test) for a comparison with MP signal without OM (n = 5).

We used a previously *ex vivo* method^5^ to measure the uptake of the odorant by the OM accounting for its metabolism. Briefly, a fresh explant of OM was placed into a glassware trap, in which a known concentration of gaseous odorant was continuously delivered; in the same time the glassware headspace was also continuously analyzed by PTR-MS instrument recording the MP signal (m/z = 85.064791) (Figs 1 and S1). The first assay was conducted with the MP at 10^−9^ g/ml continuously delivered above the OM in the glassware trap, in comparison with controls without OM or with heated OM to denaturate the enzymes present in this olfactory tissue (Fig. 2A,B). The kinetic profile of the MP signal monitored in  real-time during 1 min was significantly decreased in less than 2 sec (p < 0.01) (Fig. 2A) to reach a lower value of 18.44% (p < 0.05) at the end of the measure in presence of newborn rabbit’s OM (Fig. 2B); this corresponds to a very active metabolism provided by the OMEs present in the OM. In fact, this short delay corresponded to the time for the odorant flow to travel the dead volume of the glassware and transfer lines. As expected, the same experiment realized with heated OM instead of intact OM led to a kinetic profile not significantly different (p > 0.05) from the control in absence of OM.

When the MP was trapped above the OM during 1 min using the trapping method, a clear decrease of MP signal appeared in comparison with the control (42.85%, p < 0.001; Figs S2, S3A), which suggests an important metabolic activity. In this method, OMEs had more time to metabolize odorants, which were not continuously renewed as with the continuous method; therefore, the signal variations were increased (42.85% vs. 18.44%, respectively).

We previously showed that the MP metabolism can be inhibited by enzymatic competition with an other odorant leading to higher bioavailability of the MP and higher response of newborn rabbit to the pheromone^18^. Here, using the *ex vivo* PTR-MS method, we showed an efficient and fast metabolism olfactory metabolism toward the MP. Thus, using this method, to highlight on the function of OMEs in odorant processing, we investigated the efficiency and the velocity of the MP metabolism in the condition of odorant competition. Several assays were conducted using the trapping method to expect a modulation the MP metabolism in presence of a challenger odorant. Binary mixtures composed of MP at 10^−9^ g/ml and 2MP2, a challenger of GST previously identified^18^, were realized at various concentrations (see Supplementary Fig. S3B). For the mixture consisting in MP 10^−9^ + 2MP2 10^−9^ g/ml, the inhibition of the MP metabolism was 6.1%, i.e., there was almost no odorant-odorant interaction. Next, using increasing odorant challenger concentrations in mixture with a fixed MP concentration, we observed that the MP metabolism was inhibited in a dose dependent manner to reach a maximum inhibition of 100% for MP 10^−9^ + 2MP2 4 × 10^−6^ g/ml. These results are in line with the behavioral ones from Hanser *et al*. 2017, showing that non-reactogenic concentrations of 2MP2 (10^−9^, 10^−8^, 10^−7^, 10^−6^ g/ml) in mixture with a non-reactogenic concentration of MP (10^−9^ g/ml) induced sucking-related behavior in pups in a dose-dependent manner^18^.

### Validation of nasal mucus sampling

Here, we assessed the presence and quality of mucin (glycosylated proteins of the mucus) in our collected mucus samples by a crystallographic test. In presence of mucin, the salt crystals present in DPBS became fern-like and observation with optic microscope allowed to visualize these specific structures in a control mixture containing bovine mucin in DPBS (see Supplementary Fig. S4A). Similar conditions with the collected mucus of newborn rabbits revealed the presence of these specific fern-like structure (see Supplementary Fig. S4D). Controls realized with mucin in H_2_O (see Supplementary Fig. S4B) or collected mucus in H_2_O (see Supplementary Fig. S4E) and DPBS without mucin or mucus (see Supplementary Fig. S4C) did not present any fern-like structures. This demonstrates that our method allows a reliable sampling of the nasal mucus.

### Glutathione conjugation activity in the newborn rabbit nasal mucus

**Figure 3:**
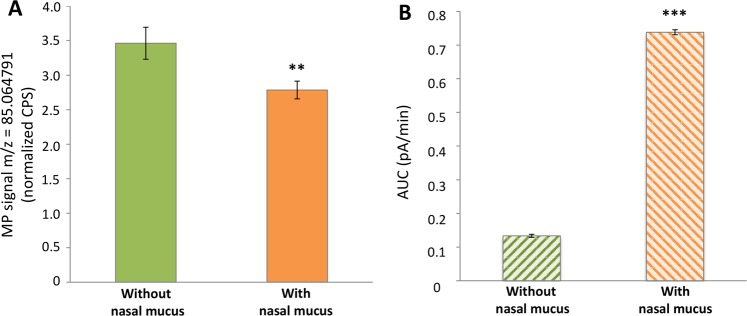
MP metabolism and enzymatic glutathione conjugation activity by nasal newborn rabbit mucus. (**A**) The MP signal in gaseous form (10^−10^ g/ml in gas bag B) passing through the glassware without nasal mucus (in green) or with nasal mucus (in orange) was monitored by the PTR-MS using the trapping method. Data represent the normalized CPS mean ± SEM during the first 90 sec of the 2 min-purge of the glassware trap of the continuous supply signal of MP measured by PTR-MS. ^**^p < 0.01 (Student’s t-test) for a comparison of the MP signal with *vs.* without nasal mucus (n ≥ 6). (**B**) Glutathione conjugation of MP by nasal mucus GST was determined by measurement with HPLC-CAD, after incubation (80 min at 37 °C) of MP + reduced glutathione in presence or absence of mucus. Bars represent the production of the glutathione-MP conjugate without nasal mucus (hatched green lines) or with nasal mucus (hatched orange lines). Data consist in the mean of 3 independent assays ± SEM. ^***^p < 0.001 (Student’s t-test) for a comparison of the glutathione-MP conjugate signal with *vs.* without nasal mucus (n ≥ 6).

Our previous works realized on the OM of newborn rabbits highlighted the glutathione conjugation of the MP by GST^16,18^ and we have shown here that this metabolism is rapid (Fig. 2A). Since the MP metabolism in the OM presents a high velocity, it seems reasonable to hypothesize that this mechanism implicates the mucus in the very first steps of the process, i.e., as soon as the odorant entered in the nasal cavity. Consequently, we determined the glutathione-MP conjugation by GST in the mucus by PTR-MS and HPLC-CAD analysis. Using our PTR-MS trapping method, a concentration of gaseous MP at 10^−10^ g/ml was trapped in the glassware containing the collected mucus. The MP signal was significantly (p < 0.01) decreased by 19.57% after exposition with the mucus (Fig. 3A) suggesting a metabolic activity toward MP in this mucus.

Subsequently, to highlight on the glutathione conjugation activity of the MP by GSTs in the nasal mucus, an *in vitro* HPLC-CAD method was used. The pooled mucus was incubated with glutathione (substrate of GSTs) and the MP was analyzed to measure the formation of glutathione-MP conjugate (total conjugate). A control without mucus was also realized to acquire the amount of the non-enzymatic conjugate due to chemical reaction between the MP and the very reactive specie glutathione. As a result, formation of glutathione-MP conjugate was 5.53 higher in presence of mucus compared to non-enzymatic conjugate (p < 0.001) (Fig. 3B), which attested for a GST activity in this mucus.

### Proteomic analysis of the newborn rabbit nasal mucus

GST belong to a superfamily of enzyme including many proteins in each family. In the human olfactory mucus, two GSTs were previously characterized: GSTA1 and GSTP1^19^. Three samples of nasal mucus from newborn rabbits were independently analyzed using peptide mass fingerprinting, involving tryptic cleavage combined with NanoLC-MSMS analysis. Three putative GSTs were identified in the newborn rabbit nasal mucus: GSTA1, GSTP1 and GSTMu1. The peptide resulting from the tryptic digestion and identified using NanoLC-MSMS are indicated in Table 1 as well as the uniprot accession number corresponding to each putative GSTs. These three putative rabbit GSTs are mammalian specific. The rabbit GSTP1, GSTA1 and GSTMu1 present 86%, 82% and 86% of identity respectively with the human orthologues. The three proteins present the characteristic Tyr catalytic amino acid previously describe for the human orthologues, suggesting that they are functional in the rabbit nasal mucus.

**Table 1:** Identification of three GSTs expressed in the newborn rabbit olfactory mucus.

Putative protein name	Uniprot accession number	Peptide Identification list Sample 1	Peptide Identification list Sample 2	Peptide Identification list Sample 3
GSTA1	Q08863		WLLAAAGEEFDEK	FMETAEDLDKLR_WLLAAAGEEFDEK
GSTMu1	P46409	FTLGLDFPNLPYLIDGTHK_MLLEYTDTSYEEK	FTLGLDFPNLPYLIDGTHK_IFVPGcLDAFPNLK_ IRVDILENQLMDNR_KYTMGDAPNYDQSK_ LTQSNAILR/MLLEYTDTSYEEK_VDILENQLMDNR	FQLVNVcYSPDFEK_FTLGLDFPNLPYLIDGTHK_ HGLcGETEEER_IFVPGcLDAFPNLK_KYTMGDA PNYDQSK_LTQSNAILR_MLLEYTDTSYEEK_ PMTLGYWDVR_YTMGDAPNYDQSK
GSTP1	G1U9R0	LQDGDLTLYQSNAILR_PPYTIVYFPVQGR_ YITLIYTNYDAGKDSYVK	LQDGDLTLYQSNAILR_MLLTDQGQSWK_ PPYTIVYFPVQGR_YITLIYTNYDAGK_YITLIYTNYDAGKDSYVK	LQDGDLTLYQSNAILR_PPYTIVYFPVQGR_ YITLIYTNYDAGKDSYVK

The table indicates the list of the three putative rabbit GSTs with their Uniprot accession number. These GSTs were identified from three different rabbit olfactory mucus. For each sample the corresponding peptides belonging to the proteins obtain after trypsin digestion are indicated.

### Nasal mucus washing and consequences on the MP perception in the newborn rabbit

By mucus washing we expected to reduce the quantity of GST in the nasal cavity and thus to increase the odorant-odorant interaction toward this enzyme, leading to a modification of the MP metabolism and *in fine* MP perception by the newborns^18^. The gel analysis of the protein content of the mucus sampling before and after mucus washing confirmed that the procedure decreases the protein concentration of the nasal mucus. The density of the band lanes of MW around 30 kDa (corresponding to GST MW) enzymes were decreased after nasal mucus washing (about 2 fold lower). We thus exposed newborns to the signal before/after washing of the mucus (see Supplementary Fig. S5).

**Figure 4:**
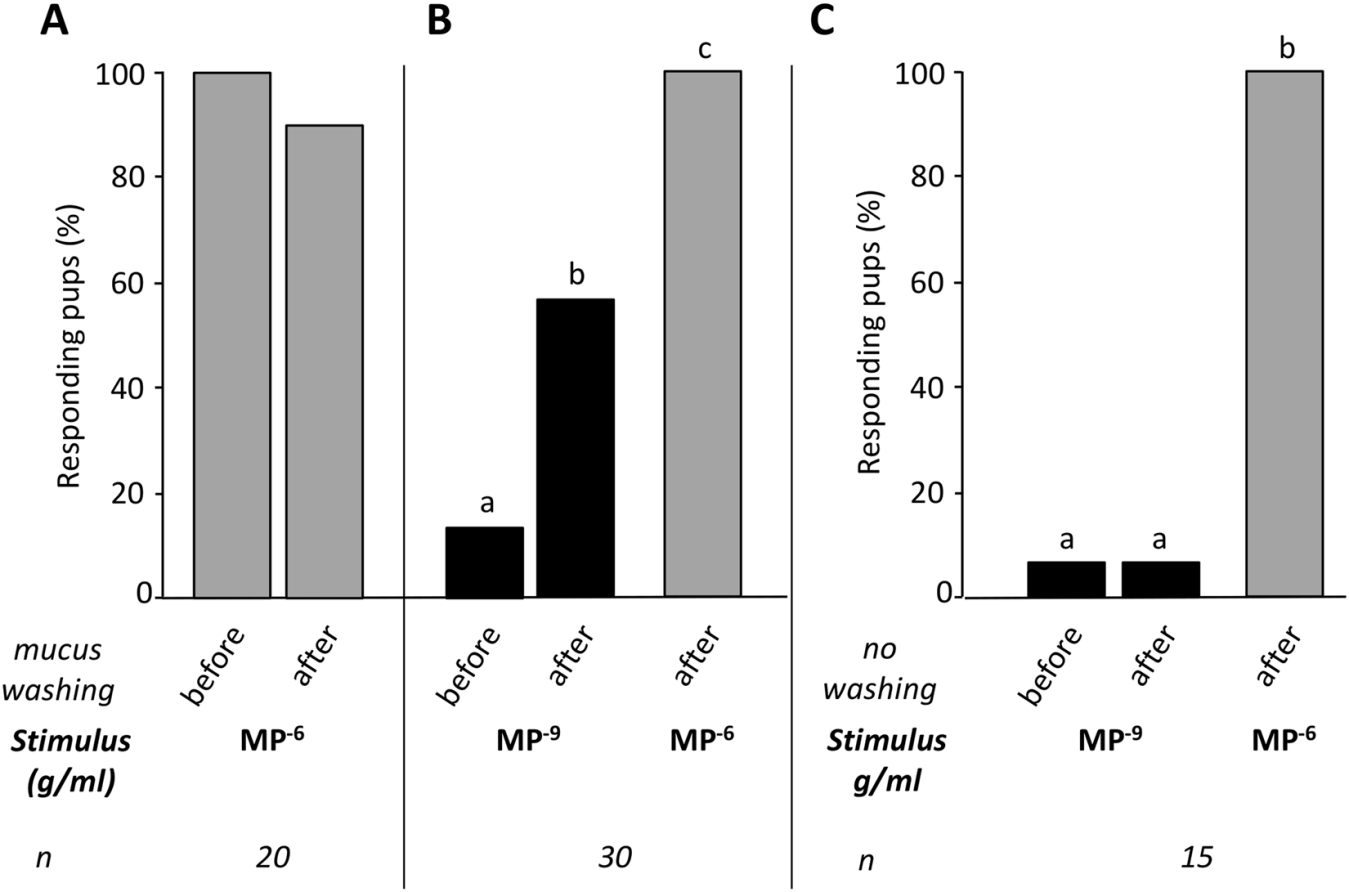
Perception and behavioral responsiveness of newborn rabbits to the MP after nasal mucus washing. Proportions of rabbit pups responding by orocephalic movements to the MP in the glass-rod test (n = 15 to 30 pups from 3–6 litters): (**A**) responsiveness to MP at 10^−6^ g/ml before and after nasal mucus washing, (**B**) responsiveness to MP at 10^−9^ g/ml before and after nasal mucus washing and subsequently presented at 10^−6^ g/ml as a control and (**C**) responsiveness to MP at 10^−9^ g/ml before and after pseudo washing and subsequently presented at 10^−6^ g/ml as a control. Within each graph, distinct digits indicate statistical differences (p ≤ 0.05): Cochran’s Q test followed by McNemar test for pairwise comparisons.

To control for the impact of the washing by itself, which could disturb the respiration of the pups and their odor-related behavior, the first assays were realized with MP alone at 10^−6^ g/ml (a fully perceptible and behavioral active concentration)^20^. Among 20 pups tested (from 4 litters), the proportion of responding pups was as strong after (90%) than before washing (100%; McNemar χ² = 0.5, p > 0.05) (Fig. 4A). Thus, the mucus washing procedure used here did not seem to modify the rabbit pups’ perception of the MP at a concentration step usually active on their sucking-related behavior.

Another group of pups (n = 30, from 6 litters) was then tested before/after washing to MP at 10^−9^ g/ml, i.e. a concentration which is just below their perception threshold (2.5 × 10^−9^ g/ml)^20^ and to MP 10^−6^ g/ml as a control after washing. The pups’ responsiveness differed according to the stimuli in these two different conditions (Q = 32.7, p < 0.001). As expected, before the washing, only a very weak proportion of pups responded to the MP (13.3%). However, after the washing, the proportion strongly increased since 56.7% of the pups displayed the orocephalic behavior (χ² = 8.64, p < 0.01); it remained lower than that induced by MP^−6^ (100%; χ² = 11.08, p < 0.001) (Fig. 4B). The increase in responsiveness to MP^−9^ was not a consequence *per se* of the second presentation of MP a few seconds after the first. Indeed, when a control group of pups (n = 15, 3 litters) was exposed to MP^−9^ two times successively without washing (pseudo-washing condition, i.e., same procedure than for the washing but with no injection or aspiration of mucus) but with the same inter-stimulation delay than the washed pups, and once to MP^−6^ at the end of the assay, these pups showed differences in responsiveness (Q = 28, p < 0.001) but almost no response to MP^−9^ both to the first then second presentation (6.7%), while they strongly responded to MP^−6^ (100%; comparison MP^−6^ vs. MP^−9^: χ² = 12.07, p < 0.001) (Fig. 4C). In other words, the proportion of pups responding to MP^−9^ after washing was clearly higher than after pseudo-washing (χ² = 10.42, p < 0.01).

Thus, when the nasal mucus was washed, rabbit pups became reactive to a concentration of MP usually inactive.

## Discussion

Our results constitute the first characterization of a phase II odorant-metabolizing activity in the nasal mucus of vertebrates. Such metabolism is of importance, as highlighted here, during the processing of odor cues, but certainly also for the protection of OM and brain from volatile toxic compounds.

In this study, the newborn rabbit-mammary pheromone model was chosen because of the olfactory metabolism of the MP, which was characterized and known to be involved in the termination and regulation of this signal of high biological value^16–18^. While we previously observed an efficient activity of the MP olfactory metabolism *in vitro*^16^ and *ex vivo*^17^, here, using real-time *ex vivo* headspace mass-spectrometry, we observed that this metabolism is also very fast. Moreover, the efficiency and velocity of the newborn OM metabolism toward the MP was also evidenced in the conditions of odorant-MP metabolic competition validating our previous behavioral results^18^.

These results take into account the whole enzymatic equipment of the OM explant used with those methods. Then, with regard to the high velocity of MP metabolism evidenced and the putative presence of GST in the nasal mucus^19^, we aimed to investigate GST activity and expression in this biological fluid, which receives and solubilizes odorants *prior* to their detection.

The reliability of the measurement of an enzymatic activity in a biological fluid depends on the condition of its collect, in order to avoid any contamination by the cellular content. We developed here an original method to guaranty a clean sampling of the nasal mucus. The visualization of crystallized mucin proteins in presence of specific buffers demonstrated the efficiency of the method and an acceptable degree of mucus dilution despite the sampling conditions. Accordingly, we were able to measure a MP metabolism in the neonatal mucus which seems as efficient than with the whole OM, although no quantitative comparison can be done since a pool of nasal mucus was used. Moreover, we showed that this activity involves GSTs by measuring the production of glutathione-MP conjugated metabolites during incubation of MP with mucus samples. Finally, the proteomic analysis of the nasal mucus revealed the presence of GSTA1, GSTP1 and GSTMu1, three rabbit GSTs potentially involved in MP metabolism. Accordingly, in newborn rabbit OM, the GST alpha was a class highly expressed and significantly more expressed than in the OM of recently weaned rabbits^16^. In comparison, GSTA1 (PO8263) was also identified in two proteomic studies performed in the mucus from human olfactory cleft, in addition to GSTP1 (P09211)^19,21^. As confirmed by our analysis, the mu class was described as the most expressed class of GST in vertebrates OM^22,23^. One should note that GST activity and expression (GST mu and pi classes) were previously evidenced in the rat intestinal mucus^24^. The mechanism of metabolizing enzymes secretion in the nasal mucus remains hypothetical. It could be due to the release along with the mucus from the Bowman glands, since GSTs expression was detected in their acinar cells^25^. The GSTs identified here in the nasal mucus are indeed soluble enzymes which activity is compatible with an aqueous environment like the mucus. However, for membrane bound enzymes like the microsomal class of GST as well as UGTs or CYP450, their activity in the mucus could be explained by exportation as exosomes as hypothesized previously^7^.

To catalyze the conjugation of odorants, phase II enzymes depend on the availability of their cofactor, the tripeptide glutathione in the case of GSTs. Accordingly, it has been shown by immunohistochemistry that the components of the gamma-glutamyl cycle, including glutathione, were localized at the mucociliary complex and therefore excreted in the olfactory mucus^26^. Although other OME activity than GST targeting the MP cannot be ruled out, its metabolism measured with real-time headspace technic supports the presence of glutathione in the mucus. This, as well as the proteomic results, confirm the reliability of our mucus sampling method. In sum, the nasal mucus appears as a reservoir of glutathione and GST for signal termination and toxicity protection of the OM.

Since the newborn rabbit nasal mucus showed a GST activity toward the MP, we then wondered whether changes in the composition of the mucus could affect the perception of this important chemosignal in rabbit pups. We performed *in vivo* mucus washing of the nasal cavity with phosphate buffer, suspected to remove or dilute the mucus constituents including GST enzymes. Strikingly, when a sub-threshold concentration (10^−9^ g/ml) of MP was proposed to the newborns after mucus washing, a large proportion of pups responded to the pheromone. This result strongly suggests that the washing discarded the GST from the mucus leading to the immediate accumulation of the MP, which may then reach an active concentration level and trigger the behavioral response in neonates. We previously observed a similar result when the GST-dependent metabolism of the MP was inhibited through simultaneous exposure to a another odorant competitor for the same enzymes^18^. In agreement, using electrophysiology in rats, it was shown that OM exposure to CYP chemical inhibitors led to the accumulation of odorants in the perireceptor environment and to a higher response toward the considered odorant^4^. Finally, in a recent study in mice, the response toward acetophenone, measured by *in situ* immunofluorescence analysis for phosphorylated S6 (pS6), appeared affected by nose washing with phosphate buffer, hypothesizing that the cytochrome P450 (CYP) enzymes involved in the odorant metabolism were removed by the washing^7^. The response did not increase as in our study because in the work by Asakawa *et al*. 2017 the signal triggering the response was actually the metabolite (methyl salycilate) resulting from acetophenone CYP metabolism^7^. Besides, given the similar function of Odorant Binding Proteins (OBP) in the regulation of odorant availability^1,27,28^ and that these proteins were previously characterized in rabbit^29,30^, a role of OBP in the results observed can not be ruled out. However, as a control, we observed that after washing, newborns responded to the reactive concentration (10^−6^ g/ml) of MP as strongly (90–100%) as before washing. This result suggests that some constituents of the mucus, including OBP may be not essential to activate the receptors and to get a MP response from the newborns, at least in our experimental situation.

To conclude, our results including data from biochemistry to behavior are the first to highlight the mechanism of odorant perception enhancement resulting from the disruption of OMEs activity in the nasal mucus of a mammal. Here, OMEs contributing to this mechanism are Phase II GST enzymes, a result that emphasizes the function of these enzymes not only in signal termination but also in signal modulation. More generally, our study reveals that the OMEs content of the nasal mucus can have a direct influence on olfactory perception and associated behavior. This may have important issue regarding olfactory troubles in newborn rabbits (some pups - ≤5% - do not respond to the MP and the reason is unknown; e.g.^13^) but also in humans since OMEs content can be affected by the regulation of the mucus secretion or by the level of expression of enzymes in the mucus, which both are modulated by the physiopathological state, drug treatment or exposure to airborne xenobiotics^31–33^. Accordingly, it has been shown recently a decreased expression of GSTP (P09211) in the mucus of elderly compared to young subjects, which was correlated with a decrease of the olfactory sensitivity^21^. Further investigation is necessary to decipher the implication of OMEs from the mucus in the peripheral processing of olfactory signals, including the characterization of the active enzymes and their substrates. Regarding the rabbit species, and the highly adaptive response of the newborn to the mammary pheromone, we will focus on the characterization of the GST involved in the metabolism of the pheromonal signal constituted by 2MB2 in order to investigate the enzymatic mechanism at the molecular level.

## Methods

### Chemicals

The mammary pheromone (MP), i.e., 2-methylbut-2-enal (2MB2; CAS # 497-03-0) and the 2-methylpent-2-enal (2MP2, CAS # 623-36-9), L-glutathione reduced (CAS # 70-18-8), mucin from bovine submaxillary glands (bovine mucin, CAS # 84195-52-8) and sterile filtered Dulbecco’s Phosphate Buffered Saline (DPBS 10×) were purchased from Sigma-Aldrich (Saint-Quentin Fallavier, France).

### Animals

New-Zealand rabbits (Charles River strain, France) originated from the breeding colony of the Centre de Zootechnie (Université de Bourgogne, Dijon). Adult females and males were housed in individual cages and kept under a constant 12:12 h light:dark cycle (light on at 7:00 a.m.) with ambient air temperature maintained at 21–22 °C. Water and food (Lapin Elevage #110, Safe, France) were provided *ad libitum*. Two days before the expected day of parturition, a nest-box (0.39 × 0.25 × 0.32 m) was fixed to the cages of pregnant females. The day of delivery was considered postnatal day 0. To even out pup-female interaction, females’ access to the nest was allowed for 15 min per day at 11:30 a.m. (this procedure allowed mimicking the short daily nursing displayed by rabbit females)^34^. We used 140 newborn rabbits on postnatal day 1 or day 2.

Local, institutional and national rules regarding the care and experimental uses of the animals were strictly followed. Thus, all experiments were conducted in accordance with ethical rules enforced by French law and approved by both the Ethical Committee of the University of Burgundy (Comité d’Ethique de l′Expérimentation Animale Grand Campus Dijon; C2EA grand campus Dijon N° 105) and French Ministère de l′Education Nationale, de l′Enseignement Supérieur et de la Recherche (MNESR) under the no. 001273.01.

### Collecting of olfactory mucosa (OM) samples

After animal decapitation, careful OM dissection was performed to avoid contamination with respiratory epithelium. The freshly removed newborn OM was immediately placed convolution upwards in the glassware trap for Proton Transfert Reaction - Mass Spectrometry (PTR-MS) experiments. The time between dissection and analyses was kept as short as possible to reduce the loss of metabolism activity.

### Collecting of nasal mucus samples

The nasal mucus was collected using an innovative device specially developed for this purpose. After animal decapitation, lower jaw dissection was performed to limit the deglutition movements and 20 µl of sterile filtered DPBS 1 × (DPBS 10 × diluted at 1/10 with ultrapure water), at pH = 7, were introduced in each nostril to increase the mucus volume and facilitate the next aspiration step. The nasal mucus was then carefully drawn through a sterile capillary tubing (0.9 mm internal diameter, 130 cm length) using a homemade reverse automatic syringe pump set to 1 ml/min. For PTR-MS experiment, the mucus of 18 newborn rabbits was pooled, and immediately placed into ice before measurements to avoid reduction of metabolic activity. For HPLC-CAD experiments, mucus of 15 newborn rabbits was pooled and the protein content was measured using microvolume NanoDrop spectrophotometer ND 1000 technology before storage at −20 °C.

### Validation of mucus sampling

It has been shown that drying of mucosal fluids in presence of Na and K ions forms fern-like patterns due to crystallization of mucins (glycosylated proteins) which are easily observable by optical microscopy (Fern-Test). We applied a similar method to the nasal mucus to validate our sampling method by confirming the presence of mucin and by evaluating its quality. A pool at 2 g/l of the mucus of 10 newborn rabbits was sampled and diluted at 1/10 in DPBS x1 or H_2_O (negative control) and were deposited on a glass slide (3 × 5 µl drops) and controls with bovine mucin at 2 g/l, diluted at 1/10 in DPBS x 1 or H_2_O, were also realized. Another control with only DPBS x 1 (no mucus) was carried out in the same conditions. After about 4 h of drying at 37 °C, the slides were examined with a microscope Eclipse E600 equipped with a 4x plan fluor objective. Images (see Supplementary Fig. S4) were acquired with a Ds-Ri2 digital camera using the software Nis-Elements Basic Research (all from Nikon, Tokyo, Japan). The absence of blood in the samples was checked using NanoDrop spectrophotometer ND 1000 technology.

#### PTR-ToF-MS (PTR-MS)

The Proton Transfert-ReactionTime of Flight-Mass Spectrometry (PTR-ToF-MS) instrument (PTR-ToF-MS 8000, IoniconAnalytik, Innsbruck, Austria) was coupled with an enclosed thermostated system developed to analyze the real-time metabolic capacity of *ex vivo* OM or nasal mucus^5^. The developed instrumentation consisted in an oven-thermostated (30 °C) circuit, containing a glassware trap hermetically closed with a Rotulex® system whose end was connected to the PTR-MS instrument (Fig. 1). The glassware trap received the freshly dissected OMs or nasal mucus deposited in two graphite cups (50 µl of mucus/cup). A stainless-still strainer was installed at the entrance of the trap to spread out the air flow. The complete assembly was connected to the PTR-MS instrument with a heated transfer line maintained at 110 °C to prevent condensation phenomena. The flow rate entering the PTR-MS instrument was fixed at 160 ml/min. The gas flows were delivered to the system from Tedlar® gas bags (#GSTP016-1818S with screw cap, Jensen Inert Products) located outside the oven, at the temperature of the air-conditioned room (22 ± 1 °C) and were inflated with 15 l with zero-air. The first one (gas bag A in Fig. 1) contained 250 ml of ultrapure water (milli-Q® system, Millipore, Molsheim France) to allow delivery of humidified air to maintain a constant moisture in the system. The second one (gas bag B in Fig. 1) contained 250 ml of an aqueous solution of MP, 2MP2 or mixtures of these odorants. The air-water partition coefficients of the MP and 2MP2 are respectively 0.0065^17^ and 0.0099^18^ respectively. These values allowed determining their respective concentrations in aqueous and gas phases. The third one (gas bag C in Fig. 1) contained 250 ml of ultrapure water. The flow from gas bags A and B passing through the glassware into the oven thermostated before being analyzed by the PTR-MS instrument. The flow from the gas bag C was directly analyzed by the PTR-MS without passing through the oven. All the gas bags were prepared the day before the experimentation to ensure headspace equilibrium.

Data were recorded with the TOF-DAQ software in the range m/z 0 to 250 at one spectrum per 0.499 second. The mass calibration was realized following the peaks of known ions (H_3_^18^O^+^, m/z = 21.022086; NO^+^, m/z = 29.997440; cluster (H_2_O)_2_- H_3_^18^O^+^, m/z = 57.043216) present at any time in spectra. During the acquisition we focused on specific masses: m/z = 85.064791 for the MP and m/z = 99.080441 for its potential competitor 2MP2. All analyses were carried out with a drift tube pressure at 2.3 mbar, drift tube temperature at 80 °C and a drift voltage of 486 V giving an electric field strength to number density ratio E/N of 112 Townsend (1 Townsend = 10^−17^.cm^2^.V).

#### PTR-MS on-line measurements

Data were recorded using two complementary methods. First (see Supplementary Fig. S1), odorants were continuously delivered above the sample to measure the kinetic of the olfactory metabolism. Second (see Supplementary Fig. S2), odorants were trapped in the circuit containing the sample before PTR-MS measurement. With this trapping method, OMEs had more time to metabolize odorants, which were not continuously renewed; therefore, the signal variations were increased. Before each experimental acquisition with sample for the two different methods, a control acquisition was realized without OM or with only 50 µl of DPBS/cup (mucus control) in the glassware trap. After experimental acquisition with OM, another control acquisition was realized with heated OM to confirm the enzymatic nature of the reaction. In this experimental acquisition, OM was previously heated at 80 °C for 15 min and cooled down at room temperature prior to the analysis. In order to reduce the number of pups required in that procedure, the OM samples were used for odorant metabolism measurement and then used again as heated OM for control experiments. It was previously checked that the signals obtained with OM heated after being used was not significantly different from the signals obtained with a directly heated OM^5^. Between each acquisition, the glassware trap was cleaned with ultrapure water and the circuit was dried and cleaned with zero-air to avoid the persistence of odorant molecules.

#### In a typical PTR-MS experiment with the continuous method

(see Supplementary Figure S1), the fresh sample was placed into the glassware. At the beginning of signal acquisition, humidified zero-air from the gas bag A was first flowed into the circuit to obtain the background signal level. After 1 min, the shut-off valve was opened during 1 other min to introduce gaseous odorants from the gas bag B above the OM in continuous. Then, the shut-off valve was closed during 2 min to recover the background signal level with humidified zero-air and without interrupting PTR-MS acquisition, the shut-off valve was again opened and this protocol was repeated twice.

#### In a typical PTR-MS experiment with the trapping method

(Figure S2), 1 min after background signal acquisition, the shut-off valve was opened during 1 min to introduce gaseous odorant in the circuit. Then, the shut-off valve was closed and the PTR-MS analysis was simultaneously switched on the outside gas bag C during 1 min to allow the odorant staying in contact with the sample in the glassware trap. After this trapping time, the PTR-MS analysis was redirected on the circuit containing the sample to purge the glassware trap and to measure the amount of odorant remaining. After 2 min and without interrupting PTR-MS acquisition, the shut-off valve was again opened and this protocol was repeated twice.

#### PTR-MS data analysis

All data were corrected for transmission and expressed in normalized CPS using the primary ions (m/z = 19.018 [H_3_O^+^] and m/z = 37.028 [(H_2_O)_2_H^+^]) to account for primary ions fluctuations. For each acquisition for samples and controls, results are expressed as mean of the triplicate mass spectra recording during the 20 last sec plateau of the 1 min continuous supply of odorant for the continuous method (see Supplementary Fig. S1) and during the first 90 sec of the 2 min-purge for the trapping method (see Supplementary Fig. S2). All the spectra were subtracted by the 20 sec signal recording without odorant before opening the shut-off valve (see Supplementary Figs S1 and S2) corresponding to the background signal level. For the OM and heated OM data, the mean of 5 acquisitions (corresponding to 5 rabbit dissections) was recorded. For data with mucus the mean of 6 acquisitions (from a pool of 18 pups) was recorded.

#### HPLC-CAD (Corona ultra RS Charged Aerosol Detector) assessment of enzymatic glutathione conjugation in the nasal mucus

Enzymatic incubations were carried out in a reaction mix containing 280 µl of mucus (from a pool of 15 pups) at 1.8 g of protein/l in DPBS x 1, 11 µl of L-glutathione reduced at 0.1 M dissolved in DPBS x 1, and 9 µl of MP at 2.5 M dissolved in absolute ethanol. The final incubation volume was 300 µl. After 80 min incubation at 37 °C, the reaction was stopped by adding 300 µl of a 25% CuSO_4_ solution and the incubation medium was centrifuged for 10 min at 14 000 g. The supernatants containing glutathione-MP conjugates were diluted at 1/3 with ultrapure water and they were analyzed by a high-performance liquid chromatography method (HPLC).

The HPLC analyses were performed using Ultimate 3000 series system equipped with dual low pressure gradient pump with vacuum degasser, a thermostated autosampler (set to 15 °C), a thermostated column compartment (set to 30 °C) and Corona Ultra RS Charged Aerosol Detector (CAD; Thermo Scientific Dionex, France). Nitrogen gas from nitrogen generator NM30LA (LGS, France), regulated at 35 psi, was introduced into the detector and the resultant gas flow was regulated automatically and monitored by the CAD device. Nebulizer temperature was set to 14 °C. Response range was set to 100 pA full scale.

The reversed phase HPLC of glutathione-MP conjugates analysis was performed on Hypersil^®^ GOLD C18 analytical column (150 mm, 2.1 mm; 3 µm particule size; ThermoScientific, France) using a multistep gradient with (A) 0.1% trifluoroacetic acid (TFA) in ultrapure water and (B) 0.1% TFA in methanol as mobile phase. Gradient elution began at 99.5% (A) and 0.5% (B). It was kept constant for 6 min, increased to reach 60% (A) and 40% (B) at 9 min, kept constant for 3 min, and then reduced to reach 99.5% (A) and 0.5% (B) at 15 min during 5 min. The flow rate of the mobile phase was set at 0.6 ml/min during the 20 min analysis time and the injection volume was 5 µl of samples. Data processing was carried out with Chromeleon 7.2 software (Dionex, France) and peak area corresponding to glutathione-MP conjugate was integrated. To determine the part of enzymatic glutathione conjugation of MP, HPLC quantifications of the MP conjugates were systematically performed in presence and absence of nasal mucus, two conditions representing respectively the total conjugation (enzymatic and non-enzymatic) and the non-enzymatic conjugation. Enzymatic glutathione conjugation was defined by subtracting the non-enzymatic conjugation part from total conjugation.

#### SDS-PAGE

5 µl of nasal mucus before and after washing was mixed with a solution containing Tris 150 mM, pH 7.0; DTT (250 mM); SDS (12%), bromophenol blue (0.04%) and glycerol (30%) and loaded on a SDS-PAGE 12%. The mucus of 6 newborn rabbits was pooled for each condition.

#### NanoLC-MSMS analysis

The nasal mucus of 12 newborn rabbit was collected in 3 tubes as describe in “**Collecting of nasal mucus samples”**. After collecting, the three samples were centrifuged at 20 000 g during 20 min at 4 °C. Then each supernatant was digested using 200 ng of trypsin overnight. Peptides were automatically fractionated onto a commercial C18 reversed phase column (75 µm × 250 mm, 2 µm particle, PepMap100 RSLC column, Thermo Fisher Scientific) and analyzed by Q-Exactive MSMS instruments (Thermo Fisher Scientific). MS/MS data was interpreted using search engine Mascot (version 2.4.0, Matrix Science, London, UK). Searches were performed with a tolerance on mass measurement of 0.2 Da for precursor and 0.2 Da for fragment ions, against a composite targetdecoy database (46172 total entries) built with a *Oryctolagus cuniculus* UniProt database (taxo 9986, May 2016, 22968 entries) fused with the recombinant trypsin and a list of classical contaminants (118 entries). Cysteine carbamidomethylation, methionine oxidation, protein N-terminal acetylation and cysteine propionamidation were searched as variable modifications. Up to one trypsin missed cleavage were allowed. For each sample, peptides were filtered out according to the cutoff set for proteins hits with 1 or more peptides taller than 9 residues, ion score >20, identity score >6, corresponding to a 1% false positive rate.

#### Behavioral assays and mucus washing

65 pups were submitted to the oral activation test, largely validated in previous studies^11,15,18,20,35,36^. It consists in the individual testing of neonates through a 10-sec presentation of a glass rod (20 cm long, 0.4 cm in diameter) carrying one of the stimuli, right under the pup nares. A stimulus was considered active when it released the typical head-searching movements (vigorous, low amplitude horizontal and vertical scanning movements of the head displayed after stretching movements towards the rod) usually followed by grasping movements (labial seizing of the rod extremity); these movements are usually displayed by rabbit newborns when contacting the mother’s abdomen and looking for the nipples during the daily nursing. Conversely, a stimulus was considered as inactive when it elicited no other response than sniffing. To minimize litter effects, each main experimental group was drawn from 3 to 6 litters, with a maximum of 5 pups/litter/group. Each pup participated in only one experiment but was successively tested with 1 or 2 stimuli. MP alone was used at two concentrations: 10^−6^ g/ml, which is maximally active on neonatal orocephalic behavior, and 10^−9^ g/ml, which is just below the perception threshold of the molecule^20^. All stimuli were diluted in ultrapure water. The pups were immediately reintroduced to the nest after testing.

For mucus washing, 100 µl of DPBS x 1 were carefully introduced by an experimenter with a tip through one nostril of a pup, gently maintained by a second experimenter. DPBS flows freely through the two nares to allow the dilution of the enzymatic concentration and cleaning of the mucus in the nasal cavity. Finally, the diluted mucus was aspired and eliminated with the same tip. The nares entrances of the pup were then dried before the presentation of stimuli. A negative control without washing consisted in a pseudo-washing, i.e. same procedure but without injection of DPBS or aspiration. If a pup responded to a stimulus, its muzzle was again softly dried before the next stimulation. When distinct stimuli were tested in the same pups, the MP at 10^−6^ g/ml was systematically presented in last position as a positive control.

### Statistics

For PTR-MS statistical analyses, data were analyzed using the Student’s t-test to compare the acquisition signals obtained in presence of OM, heated OM or mucus with the control acquisition signals without OM or without mucus. Data are expressed as means ± SEM (Standard Error of the Mean).

For HPLC statistical analyses, data are expressed as means ± SEM and the total glutathione-MP conjugate was compared with the non-enzymatic conjugate using the Student’s t-test.

For behavioral assays, the proportions of pups responding to the stimuli were compared using the χ² test of Pearson when the groups were independent (i.e., distinct groups tested for their response to a same stimulus) or the Cochran’s Q test when the groups were dependent (i.e., pups from a same group tested for their response to distinct stimuli). When the Cochran’s Q or χ² tests were significant, McNemar or χ² tests were done respectively for pairwise comparisons.

## Supplementary information

Supplementary dataset
