# Publisher Correction: Nasal mucus glutathione transferase activity and impact on olfactory perception and neonatal behavior

**DOI:** 10.1038/s41598-019-44135-0

**Published:** 2019-05-28

**Authors:** Aline Robert-Hazotte, Philippe Faure, Fabrice Neiers, Catherine Potin, Yves Artur, Gérard Coureaud, Jean-Marie Heydel

**Affiliations:** 1 Centre des Sciences du Goût et de l′Alimentation, UMR 6265 CNRS/1324 INRA/Université de Bourgogne Franche-Comté, 9 boulevard Jeanne d’Arc, F-21000 Dijon, France; 2 Centre de Recherche en Neurosciences de Lyon, INSERM U1028/CNRS UMR 5292/Université Lyon 1, CH Le Vinatier-Bâtiment 462 Neurocampus, 95 boulevard Pinel, F-69675 Bron, France

Correction to: *Scientific Reports* 10.1038/s41598-019-39495-6, published online 28 February 2019

The original version of this Article was published with an incorrect version of the Supplementary Information file due to a technical error. This has now been corrected in the HTML; the PDF version of the paper was correct from the time of publication.

