# Supplementary material for: Nasal mucus glutathione transferase activity and impact on olfactory perception and neonatal behavior
Source: Sci Rep. 2019 Feb 28;9:3104. doi: 10.1038/s41598-019-39495-6 (PMC6395716; doi:10.1038/s41598-019-39495-6)
Supplement: Supplementary file 1 — Supplementary dataset [file 41598_2019_39495_MOESM1_ESM.pdf]

Nasal mucus glutathione transferase activity and impact on olfactory perception and neonatal behavior

Aline Robert-Hazotte, Philippe Faure, Fabrice Neiers, Catherine Potin, Yves Artur, Gérard Coureaud & Jean-Marie Heydel

A

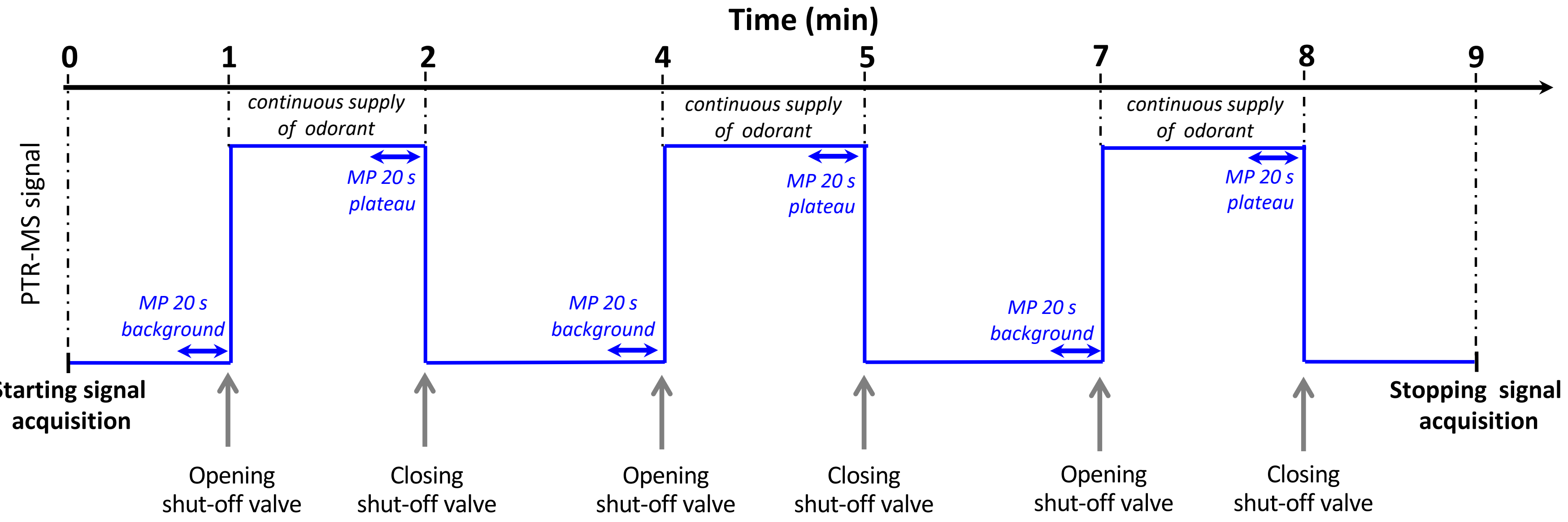

Figure S1: Schematic representation of the protocol used for real-time on-line measurements of the *ex vivo* MP metabolism by PTR-MS with the continuous method.

At the start of the signal acquisition, the PTR-MS measured the device background signal. The opening of shut-off valve without interrupting PTR-MS acquisition resulted in an immediate and characteristic increase of the continuous MP signal passing through the glassware containing or not samples during 1 min. The closure of the shut-off valve allows to recover the background signal level and this protocol was repeated twice to record triplicate values for MP and background signals to obtain the fair evaluation of the MP metabolism.

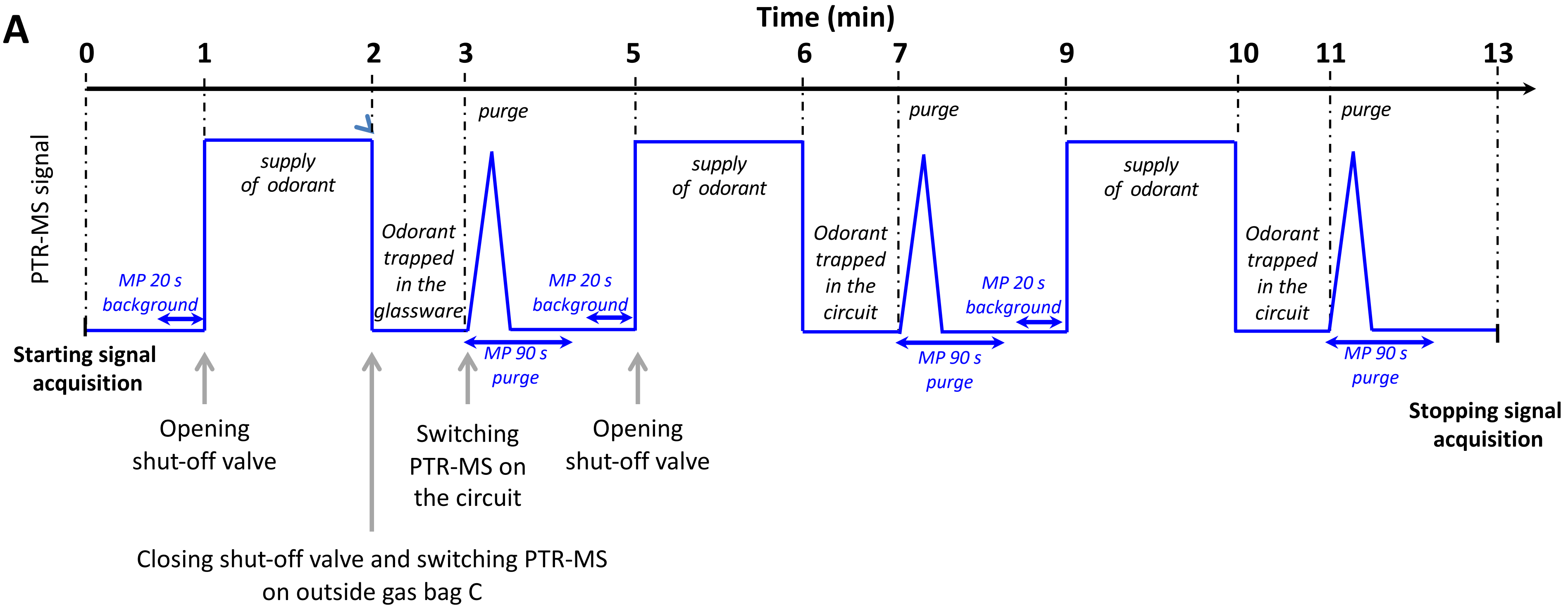

Figure S2: Schematic representation of the protocol used for real-time on-line measurements of the *ex vivo* MP metabolism by PTR-MS with the trapping method.
(A) At the start of the signal acquisition, the PTR-MS measured the device background signal. The shut-off valve was opened to introduce gaseous odorant in the glassware. Then, the shut-off valve was closed and the PTR-MS analysis was simultaneously switched on the outside gas bag to allow the trapping of the odorant into the glassware. After 1 min the PTR-MS analysis was redirected on the circuit containing the glassware to purge the trap to measure the amount of odorant remaining and to recover the background signal level. This protocol was repeated twice to record triplicate values for MP and background signals to obtain the fair evaluation of the MP metabolism.

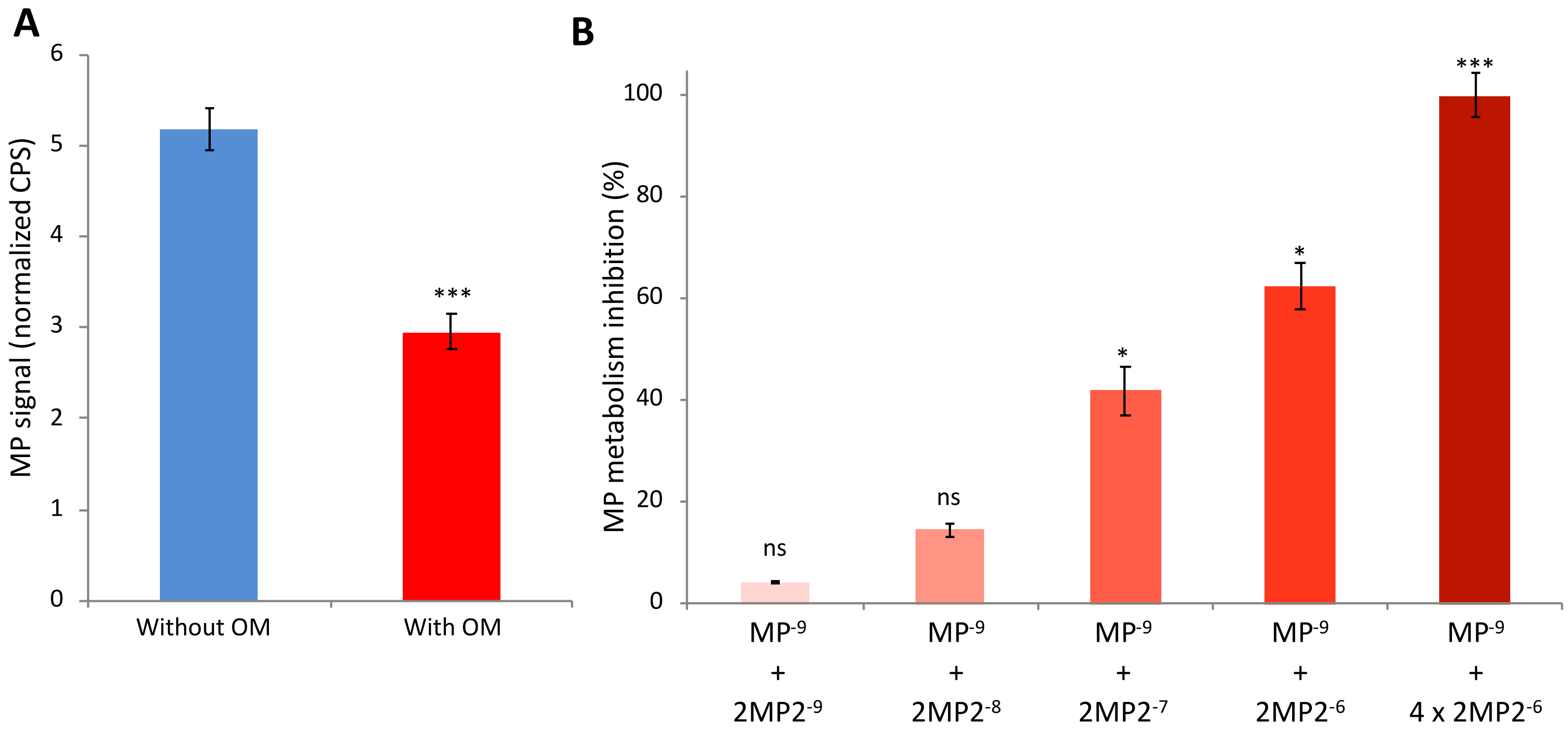

Figure S3: Real-time *ex vivo* newborn rabbit OM metabolism of the MP by PTR-MS analysis with the trapping method in presence of various amount of a challenger odorant, 2MP2.
The signal of MP on gaseous form (10^{-9} g/ml in gas bag B) passing through the glassware without OM (in blue) and with OM (in red) was monitored in real time by the PTR-MS using the trapping method. Data represent (**A**) the normalized CPS mean ± SEM during the first 90 sec of the 2 min purge of the glassware trap of MP signal measured by the PTR-MS instrument. ***: $p<0.001$ (Student's t-test) for a comparison of the MP signal with vs. without OM ($n = 5$). (**B**) the % of MP metabolism inhibition in presence of various amount of 2M2P. Results are expressed as % of MP signal measured by PTR-MS during the first 90 sec of the 2 min purge of the glassware trap in presence of 2MP2 compared to the signal obtained for the MP alone (control). The % are means of $n = 3$ replicated measures ± SEM. ns: no significant difference; * and ***: differences at $p \leq 0.05$ and $p \leq 0.001$, respectively, between the control condition (MP alone) and mixture conditions (Student's t-test).

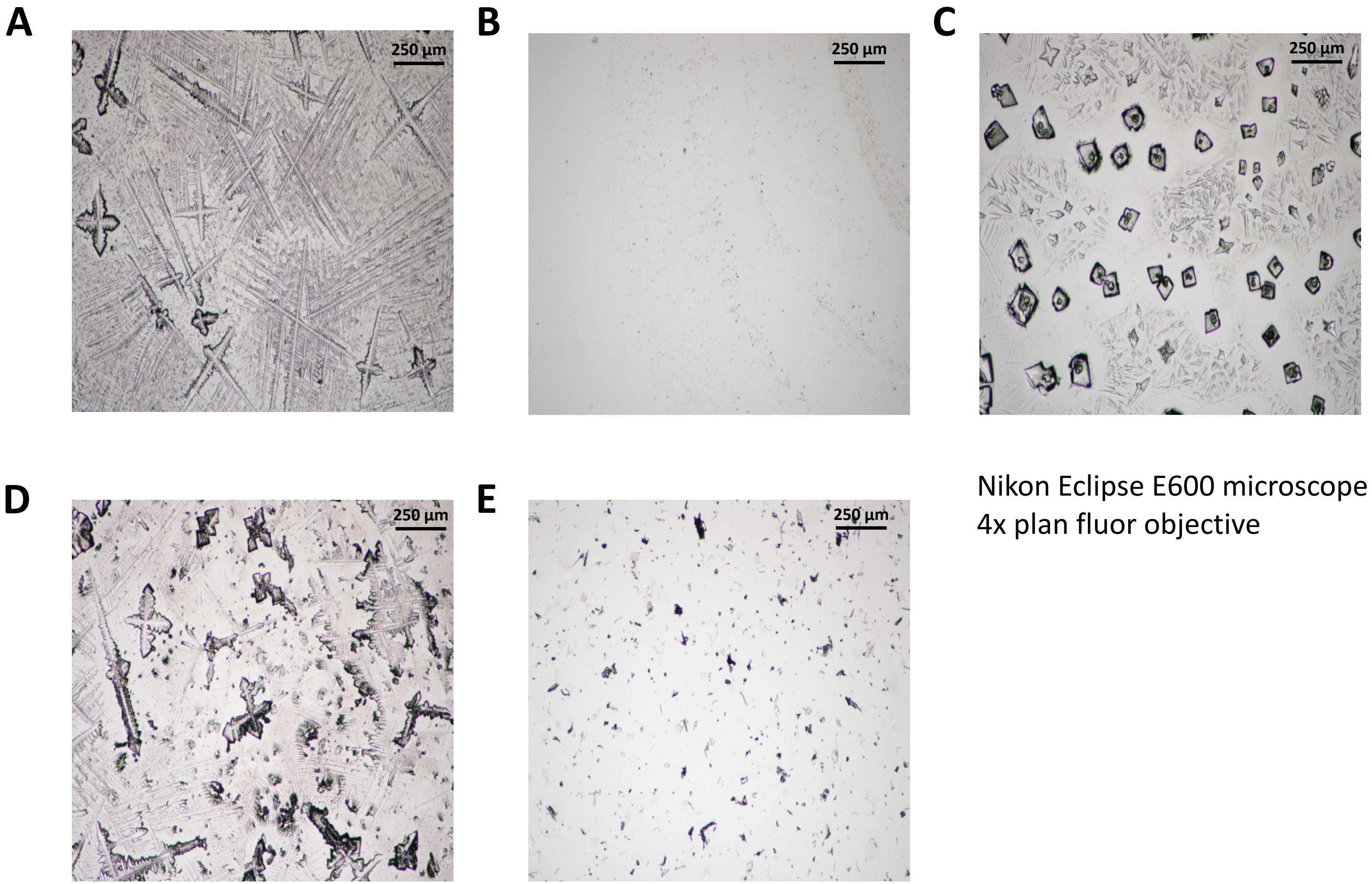

Figure S4: Evaluation of mucus samples by the Fern-Test from newborn rabbits.
Microscopy images corresponding to (**A**) bovine mucin (2g/l) diluted at 1/10 in DPBS x 1 or (**B**) H_2O, (**C**) DPBS X 1 alone and (**D**) newborn rabbits collected mucus (2g/l) diluted at 1/10 in DPBS x 1 or (E) H_2O. All slides were examined with a microscope Eclipse E600 equipped with a 4X plan fluor objective. Images were acquired with a Ds-Ri2 digital camera using the software Nis-Elements Basic Research (all from Nikon, Tokyo, Japan). Scale bar corresponds to 250 µm.

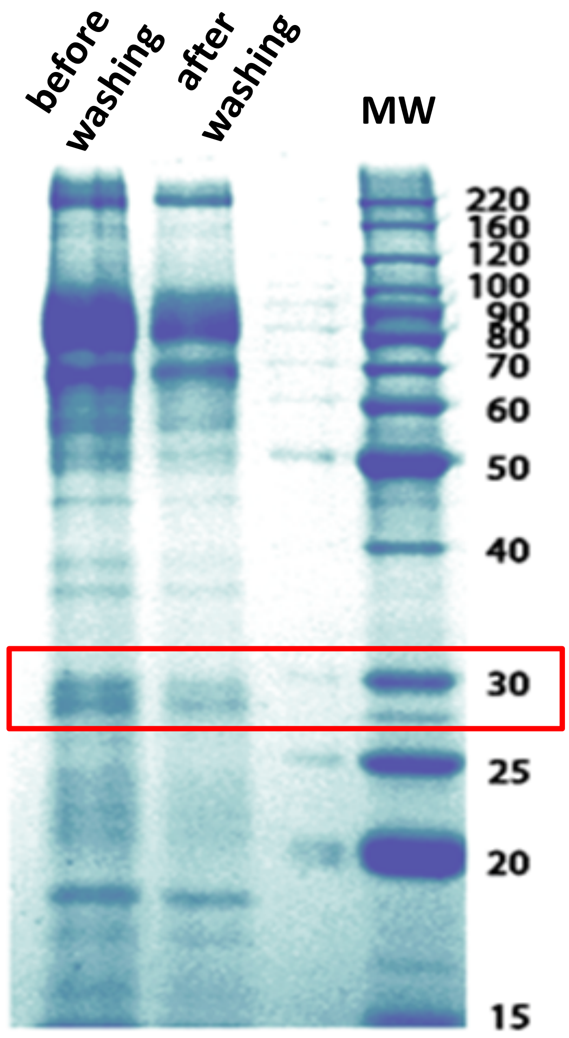

Figure S5 : Evaluation of the nasal mucus washing efficiency

Mucus washing efficiency was evaluated by SDS-PAGE obtained with a 5 µl deposit of newborn rabbits nasal mucus collected before and after nasal washing (n=6 pooled samples for each condition). A full-length unprocessed gel image is presented. The band lanes of MW around 30 kDa include GST enzymes.
